# A Novel Minimally Invasive Surgically Induced Skeletal Muscle Injury Model in Sheep

**DOI:** 10.3390/ijms25115612

**Published:** 2024-05-21

**Authors:** Laura Vidal, Ingrid Vila, Vanesa Venegas, Anabel Sacristán, Paola Contreras-Muñoz, Maria Lopez-Garzon, Carles Giné, Gil Rodas, Mario Marotta

**Affiliations:** 1 Leitat Technological Center, Carrer de la Innovació 2, 08225 Terrassa, Spain; 2 Bioengineering, Cell Therapy and Surgery in Congenital Malformations Laboratory, Vall d’Hebron Institut de Recerca (VHIR), Universitat Autònoma de Barcelona (UAB), 08035 Barcelona, Spain; 3 Medical Department of Futbol Club Barcelona (FIFA Medical Center of Excellence) and Barça Innovation, 08970 Sant Joan Despí, Spain; 4 Sports Medicine Unit, Hospital Clínic and Sant Joan de Déu, 08950 Barcelona, Spain; 5 Faculty of Medicine and Health Sciences, University of Barcelona, 08007 Barcelona, Spain

**Keywords:** animal experimentation, athletic injuries, biopsy needle, soft tissue injuries, magnetic resonance imaging, muscle rupture, sheep, skeletal muscle

## Abstract

Sports-related muscle injuries account for 10–55% of all injuries, which is a growing concern, especially given the aging world population. To evaluate the process of skeletal muscle injury and compare it with muscle lesions observed in humans, we developed a novel in vivo model in sheep. In this model, muscle injury was induced by an ultrasound-guided transverse biopsy at the myotendinous junction of the medial gastrocnemius muscle. Twelve male sheep were examined at 3, 7, 14, and 28 days post-injury. Histological, immunofluorescence, and MRI analyses indicate that our sheep model could resemble key human clinicopathological features. Statistically significant differences (*p* < 0.05) were observed in collagen I, dMHC, α-SMA, and CD68 immunohistochemical detection when comparing injured and healthy muscles. The injured gastrocnemius muscle exhibited elevated levels of type I collagen, infiltration of CD68(+) macrophages, angiogenesis, and the emergence of newly regenerated dMHC(+) myofibers, which persisted for up to 4 weeks post-injury. Similarly, the progression of muscle injury in the sheep model was assessed using advanced clinical 3 T MRI and compared with MRI scans from human patients. The data indicate that the sheep muscle injury model presents features similar to those observed in human skeletal muscle injuries. This makes it a valuable large animal model for studying muscle injuries and developing novel therapeutic strategies.

## 1. Introduction

Skeletal muscle is one of the most abundant tissues in the human body, constituting approximately 40–45% of total body mass [1,2,3]. It plays a crucial role in sustaining life by contributing to mobility, joint stability, postural control, breathing, metabolic control, thermoregulation, energy storage, and more [1,4]. For that reason, the skeletal muscle is constantly exposed to various types of injuries. Athletes, both professional and amateur, are at an elevated risk of skeletal muscle injuries due to their increased physical activity levels and the high number of practice hours per week [5,6]. Additionally, the risk is higher for older athletes and those with a history of previous muscle injuries [7]. Muscle injuries account for 10–55% of all sports-related injuries [8,9]. Furthermore, the progressive aging of the population can make these injuries a major social and health problem due to muscle loss and locomotor problems, which particularly affect older people [10].

Skeletal muscle injuries encompass a broad range of causes and severities, including indirect injuries such as strains, direct injuries such as contusions or lacerations, and degenerative diseases such as muscular dystrophies [2,3,11]. Contusions and strains are responsible for 90% of sports injuries, while lacerations are much less common [2,8,9]. Specifically in strains, the inability of muscle fibers to sustain the demands of excessive exercise and the overstretching of myofibrils potentially results in their rupture [2,9]. This muscle-contraction-induced injury occurs mainly due to intense eccentric contractions or overstretching of the muscle. It is usually associated with dynamic, non-contact sporting activities such as sprinting and jumping and can be more complicated to treat if the tear occurs at the myotendinous junction, as is often the case with lower extremity injuries [2,7].

Managing muscle injuries in sports can be challenging due to limited scientific evidence and inconsistent categorization. Recent proposals for classifying and grading these injuries, although validated for hamstring injuries, lack clarity in their applicability to other muscles [12]. Common elements across classifications emphasize considering anatomical location, connective tissue usage, and describing injury patterns [5,13,14,15]. The grading classification is primarily based on radiologic features assessed through ultrasound (US) and magnetic resonance imaging (MRI). It focuses on evaluating the injured connective tissue without specific emphasis on edema quantification [16]. In 2020, Pedret et al. [17] proposed a sonography-based classification for distal gastrocnemius injuries, identifying four types based on lesion location and radiographic signs. The anatomy of the distal triceps surae has a unique influence on injury patterns within the myoconnective complex. These patterns can be detected through fluid-sensitive sequences that reveal fiber disruption, aponeurosis discontinuity, hematoma extension, and muscular asynchrony.

The overall process of muscle injury and repair occurs regardless of the type or severity of muscle injury and follows three phases [3,4,11,18]: destruction, repair, and remodeling. During the destruction phase, myofibers and surrounding blood vessels experience rupture and necrosis, leading to hematoma formation and an inflammatory response that activates macrophage-mediated phagocytosis. In the second repair phase, necrotized tissue undergoes phagocytosis, producing a connective tissue scar. Capillary ingrowth into the injured area, activation of satellite cells, and subsequent differentiation and proliferation of myoblasts occur, generating new myofibers that regenerate and repopulate the damaged muscle tissue. This phase involves the crucial roles of pro-inflammatory M1 and anti-inflammatory M2 macrophages. M1 macrophages remove debris, degrade structural components, and secrete cytokines that promote the proliferation and migration of satellite cells. The timely switch to M2 macrophages stimulates the fusion of myoblasts into myotubes while inhibiting excessive extracellular matrix (ECM) deposition by fibrogenic/adipogenic progenitors. Myotubes form within a few days, replacing the necrotized part of the ruptured myofiber and penetrating the connective tissue scar. Finally, the third remodeling phase, closely associated with the repair phase, focuses on the maturation of regenerating myofibers, the remodeling of the ECM in the connective tissue, the reorganization of scar formation, and the establishment of neuromuscular junctions to restore the muscle’s functional capacity. 

However, the process of muscle regeneration often fails to fully restore the muscle to its pre-injury functional state. This failure in restoring the muscle’s functional state can be attributed to the loss of contractile tissue and the formation of fibrotic scar tissue, which can lead to significant physical impairment, pain, and discomfort for the patient [4]. Muscle regeneration is frequently studied using in vivo models in small animals, which require the induction of muscle damage. Common methods [19] to produce muscle injury include the use of myotoxic or chemical agents, such as cardiotoxin [20] or barium chloride [21], and physical procedures such as eccentric contractions [22], freeze injury [23], electroporation [24], contusion [25], or surgically induced controlled and localized muscle injuries [26]. Less commonly, in vivo studies are conducted in large animals, such as the sheep model, due to the anatomical and physiological characteristics that they share with humans [27]. This approach provides valuable translational insights into disease progression and therapeutic mechanisms, which are crucial for the development of future human therapies. The sheep model is an excellent large animal model for studying the musculoskeletal system. It closely mimics the distribution of mechanical loads across joints during weight-bearing activities observed in humans, making it a highly comparable and translational model [27]. The sheep model has been previously used to study muscle injuries, such as volumetric muscle loss (VML) and grade II muscle lacerations [28,29]. Although these types of injury models, involving complete muscle dissection or surgical laceration, can provide a reproducible muscle injury, they require the use of highly invasive surgical approaches and can cause severe damage to adjacent tissues.

This study presents a new in vivo model of surgically induced minimally invasive muscle injury in large animals. The model aims to evaluate the process of skeletal muscle lesion and regeneration in the sheep and compare it with muscle lesions observed in humans. This experimental model was developed in sheep and is based on a previous experimental model generated in rats [30], where a commercially available biopsy gun was used to create skeletal muscle injuries in an individual muscle or a single muscle group through a minimally invasive surgical approach.

## 2. Results

### 2.1. Surgically Induced Skeletal Muscle Injury in a Sheep Model

**Figure 1:**
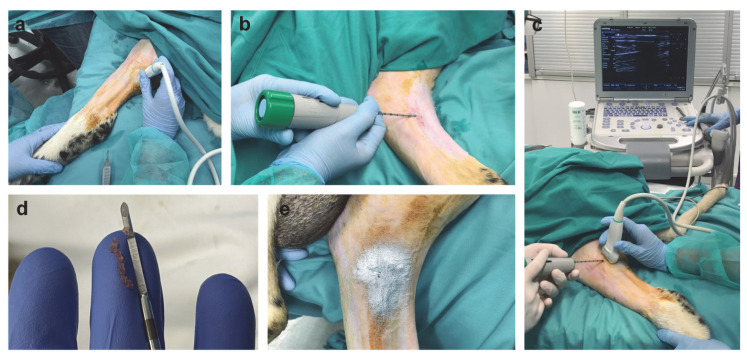
Minimally invasive surgically induced muscle injury model in sheep. (**a**) Animals were anesthetized and placed in right lateral decubitus position prior to surgery, and the myotendinous junction of the gastrocnemius muscle was localized using US. (**b**) Skeletal muscle injury in the sheep was generated using a 12 G biopsy needle. (**c**) US confirmation of the location of the biopsy needle within the gastrocnemius muscle and surgical follow-up. (**d**) Obtention of the muscle biopsy. (**e**) Postoperative wound treatment with aluminum spray as an external antiseptic to ensure good healing of the wound.

The surgical procedure for inducing muscle injury in the gastrocnemius muscle of the sheep is outlined in Figure 1. US was used to examine the musculature of the sheep’s leg and identify the location of the myotendinous junction of the gastrocnemius muscle for generating the muscle lesion. A 12 G biopsy needle with a 4 mm diameter was then transversely inserted approximately 2.5 cm from the muscle–tendon junction. We confirmed the precise placement of the biopsy needle in the gastrocnemius muscle through US. The lesion was created by triggering the biopsy gun, which caused a transverse biopsy within the muscle, resulting in muscle fiber rupture (Supplementary Materials, Video S1). There were no complications, and all animals completed the study.

### 2.2. Histology Analysis of Skeletal Muscle Injury

**Figure 2:**
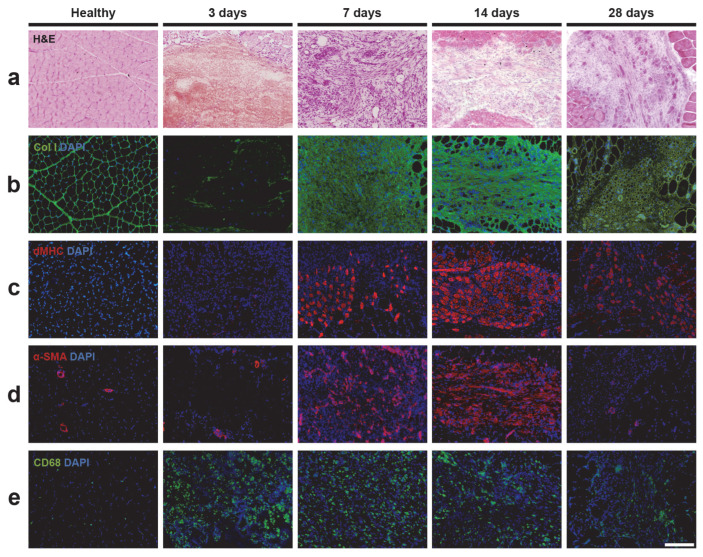
Histological and immunofluorescence analyses of the progression of the skeletal muscle injury produced in the sheep model. Representative images (N = 3 for each time point) show photomicrographs (10×) of hematoxylin and eosin (H&E)-stained (**a**) and immunofluorescence-stained muscle sections for type I collagen (**b**), dMHC (**c**), α-SMA (**d**), and CD68 (**e**) markers at different points in time (3, 7, 14, and 28 post-injury) and compared to the healthy muscle. The scale bar indicates 200 μm.

The injury in the sheep’s gastrocnemius muscle resulted in the partial rupture of muscle fibers (Figure 2a). Three days after the muscle injury, there was a noticeable destruction of the muscle fiber structure, with minimal cellular presence and the apparent formation of a blood clot exclusively in the injured area. Seven days after the injury, the affected area was surrounded by inflammatory cells. Newly formed regenerating central-nucleus myofibers were detected at this point and remained present in significant numbers 28 days after the injury.

### 2.3. Immunofluorescence Analysis of Skeletal Muscle Injury

**Figure 3:**
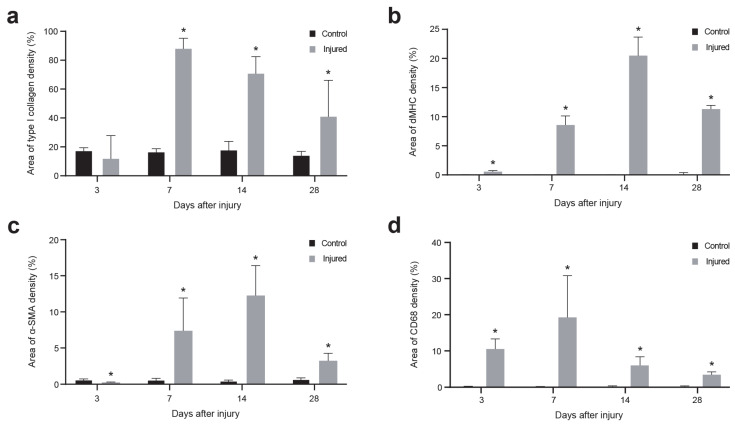
Quantitative immunohistochemical analysis of fibrosis, muscle regeneration, neovascularization, and inflammation markers to assess the progression of muscle injury. Values of the area of type I collagen (**a**), dMHC (**b**), α-SMA (**c**), and CD68 (**d**) density (%) are represented. Values are presented as mean ± SD. * *p* < 0.05 vs. control group.

We focused on a quantitative immunohistochemical analysis through area density measurement of collagen I, the main structural component of the muscle fiber ECM and a well-known marker of fibrosis; developmental form of myosin heavy chain (dMHC), a recognized marker of muscle regeneration that identifies newly formed myofibers; α-smooth muscle actin (α-SMA), a marker of neovascularization that identifies blood vessels; and CD68, a marker of inflammatory cells (Figure 2 and Figure 3).

#### 2.3.1. Extracellular Matrix and Fibrosis

Important changes were observed in the detection of collagen I marker (Figure 2b). The structure of the muscle fiber was clearly visible in the healthy muscle tissue sections; however, 3 days after the injury, there was minimal presence of collagen I due to the rupture of muscle fibers. From 2 weeks up to a month after the injury, a significant deposition of collagen I was observed in the injured area. Figure 3a shows that the amount of collagen I was remarkably low 3 days after the injury (11.92 ± 12.91%), but substantially increased, reaching its maximum levels 7 days after the injury (88.87 ± 7.94%). By day 14 after the injury, collagen I levels progressively decreased (72.12 ± 11.11%) and remained detectable at day 28 after the injury (44.03 ± 22.18%), exceeding the collagen I levels in healthy muscle (14.45 ± 4.32%).

#### 2.3.2. Muscle Regeneration

We focused on the quantitative immunohistochemical analysis of the marker dMHC, which specifically labels the embryonic/developmental myosin isoform. This isoform is only expressed during embryonic and fetal development and is later replaced by the fast and slow isoforms in mature muscle fibers. However, the embryonic/developmental myosin isoform is also expressed in adult muscles during the muscle regeneration process after injury, specifically labeling the newly formed regenerating fibers in the injured skeletal muscle area [31] (Figure 2c). Figure 3b shows that regenerating myofibers were detectable at 3 days post-injury (0.56 ± 0.39%). Starting from 7 days after the injury, the presence of dMHC increased significantly (8.43 ± 2.97%), reaching its maximum at 14 days post-injury (20.38 ± 7.02%). At 28 days post-injury, a decline in the presence of dMHC was observed (11.18 ± 5.76%).

#### 2.3.3. Vascularization

A substantial increase in the presence of blood vessels was observed within the area of the muscle injury (Figure 2d). Figure 3c shows that α-SMA detection was low at 3 days post-injury (0.23 ± 0.18%), but significantly increased 7 days after the injury (7.52 ± 4.77%), reaching its peak at 14 days after the injury (12.17 ± 4.73%). This increase in α-SMA detection could indicate an increase in the number of blood vessels in the injured area during the repair process, where an increase in blood supply is required. At 28 days post-injury, α-SMA levels decreased (3.19 ± 1.88%), possibly indicating a reduction in the presence of blood vessels during a more advanced stage of the muscle regeneration process.

#### 2.3.4. Inflammation

Increased presence of macrophages was observed in the injured muscle using the well-described macrophage marker CD68 [32], whereas a low presence was observed in the healthy muscle (Figure 2e). Figure 3d shows a rapid and significant increase in the quantity of CD68 from 3 days after injury (10.80 ± 4.14%), reaching a maximum at 7 days after injury (17.45 ± 10.67%). This increase is probably in response to the injury and repair process of the affected area. A decrease in the percentage of CD68 was detected from 14 (5.89 ± 3.41%) to 28 days (3.35 ± 1.74%) after injury. The analysis revealed that healthy muscle consistently exhibited a low presence of the CD68 marker (0.21 ± 0.17%).

### 2.4. Magnetic Resonance Imaging Analysis of the Longitudinal Evolution of Skeletal Muscle Injury

**Figure 4:**
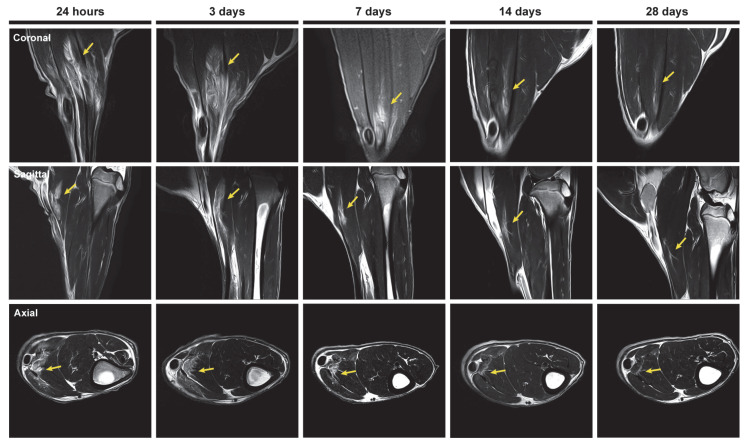
Longitudinal evolution of the sheep skeletal muscle injury by clinical 3 T MRI. Representative MRI scans of the sheep muscle injury in the coronal, sagittal, and axial planes at different time points (24 h and 3, 7, 14, and 28 days after the injury). The yellow arrows indicate the area of intramuscular edema.

We used 3 T MRI technology, an advanced and non-invasive imaging technique commonly used in human clinics that provides highly sensitive and detailed information on skeletal muscle. This allowed for precise evaluation of injury progression in the sheep model and facilitates comparison with injuries in human patients. We conducted a longitudinal MRI study in the axial, coronal, and sagittal planes to assess the evolution of muscle injury in our sheep model. The axial plane was used to evaluate muscle outlines, the myotendinous junction, and its anatomical relationship to any localized lesions, while the coronal and sagittal planes were used to evaluate the injury’s longitudinal extent [33]. Additionally, MRI is widely used to assess and follow up lower limb muscle injuries. Figure 4 shows that the surgically induced muscle injury in the sheep resulted in a partial rupture of the muscle fibers. The injury also caused strong and localized edema, which was detectable 3 days after the injury. Interestingly, we observed the distinctively classic feathery pattern, which is a hallmark of this type of muscle lesions. From that point on, the intramuscular edema decreased progressively from 7 days after the injury until 28 days after the injury, where it was still detected at low levels.

### 2.5. Surgically Induced Skeletal Muscle Injury in a Sheep Model Mimics Muscle Lesions Observed in Human Athletes

**Figure 5:**
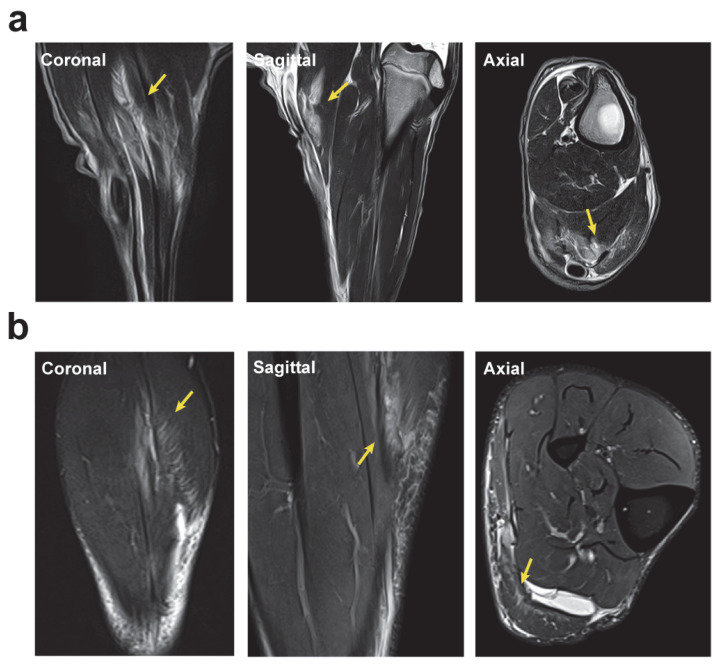
MRI in vivo imaging of the skeletal muscle injury comparison between the sheep model and a veteran human athlete. The yellow arrows indicate the area of intramuscular edema. (**a**) MRI of the sheep muscle injury model 24 h after the injury when edema with feather pattern can be observed. (**b**) MRI of a 60-year-old veteran human athlete showing the same intramuscular classical feathery pattern.

We compared 3 T MRI data to evaluate the similarities between the surgically induced muscle injury in sheep and the features observed in a veteran human athlete. Figure 5 illustrates the strong similarities between the sheep model and human injuries, which show similar clinical imaging features observed on MRI in human patients.

The MRI data depict a muscle injury in the gastrocnemius of a veteran athlete, revealing a posterior aponeurotic defect on the lateral aspect of the inner calf, accompanied by hematoma and feather edema. The free aponeurosis is compromised, causing retraction of the muscle belly. Additionally, there is an extension towards the septum with the external calf. Similarly, the sheep model’s muscle injury resulted in a detectable edema with a feathery appearance along the muscle fibers of the gastrocnemius. A small amount of fluid in the epifascial space was also observed, resembling the characteristic clinical imaging aspects of skeletal muscle injuries in athletes.

## 3. Discussion

In this study, we present a novel animal model of surgically induced muscle injury in sheep using a minimally invasive procedure with a commercially available biopsy needle. Injuries to the gastrocnemius muscle are common due to its high proportion fast-twitch type 2 fibers [34], which are highly fatigable and more susceptible to injury. Recovery periods vary depending on individual factors, and previous calf injuries increase the risk of subsequent injuries [7], which can impact muscle elasticity and contractile capacity, ultimately affecting sports activity.

This sheep model of surgically induced muscle injury may be useful in studying this type of injury and developing therapies to improve muscle regeneration. The use of large animal models in sheep to study skeletal muscle injury has been previously described, such as VML [28] and biceps femoris [29] injury models. However, their use for the study of calf injuries, which are highly prevalent among athletes and contribute negatively to their performance [35,36], has been overlooked. In our sheep muscle injury model, surgical incisions and opening tissue layers are not necessary to induce muscle injury. Instead, muscle injury is induced by transversally inserting the biopsy needle and triggering the biopsy gun. This method represents a reproducible and minimally invasive approach to generate muscle lesions.

This model of muscle injury in sheep allows for the study of histological and molecular changes that occur during the muscle injury and regeneration process. The model shows similarity between muscle lesions in sheep and those observed in human patients using state-of-the-art clinical imaging 3 T MRI analysis. The evolution of the muscle healing process in our sheep model was assessed by histological (H&E) and immunofluorescence analyses of collagen I, dMHC, α-SMA, and CD68 area density percentages as markers of fibrosis, muscle regeneration, neovascularization, and inflammation, respectively. We also used advanced in vivo MRI analysis over 4 weeks, in which the evolution of the injury and healing process was studied at different time points.

Through H&E analysis, we observed degeneration of the muscle fibers in the injured area, followed by an inflammatory phase and the appearance of newly formed muscle fibers, as has been reported in previous models inducing muscle injuries [29], and reproduces the features corresponding to the three main phases that take place in the process of muscle regeneration [3]. 

The high abundance of collagen I could indicate strong activation of the production of ECM components and the accumulation of fibrotic tissue at the injury site. Fibrosis occurs when scar tissue begins to form due to the deposition of ECM constituents, such as collagen and fibronectin, between 2–3 weeks after injury, which can decrease or increase with time [37,38]. Several growth factors, cytokines, and proteolytic enzymes are involved in fibrosis, with transforming growth factors (TGFs) being the major stimulators [37,38,39,40]. Additionally, pro-fibrotic cytokines, such as TGFs, induce the migration of fibroblasts into the wound to produce and remodel the ECM [41]. This excessive ECM deposition produced after injury, which results in muscle fibrosis, negatively affects the regeneration of muscle function, and increases susceptibility to re-injury [38,40]. The fibrotic scar produced is important for tissue integrity during the early phases of muscle healing; however, its presence in later stages has a detrimental effect on muscle function [38]. Therefore, the development of therapies aimed at reducing muscle fibrosis may be promising, such as stretching exercises [42,43,44] or the use of drugs targeting pro-fibrotic cytokines [45,46,47] that have been shown to improve muscle healing by reducing muscle fibrosis and increasing myogenesis. Several studies have shown that physical exercise and stretching have an effect on mesenchymal stem cells (MSCs) through mechanotransduction, converting the mechanical loading into different cellular responses that have an impact on improving muscle regeneration [48]. It has been observed that these stem cells present in the muscle facilitate the synthesis of new myofibers after mechanical stimuli [49] and enhance muscle repair when combined with exercise therapies [50]. It has also been observed that exercise may play a role in regulating the inflammatory response, which, in turn, promotes the proliferation and differentiation of these cells [51].

Muscle regeneration was evaluated by the presence of the embryonic/developmental myosin isoform (dMHC), which specifically labels the newly formed myofibers. Regenerating myofibers can be identified by a centrally located nucleus and exhibit a smaller cross-sectional area compared to the surrounding healthy muscle fibers. Most studies on muscle regeneration have been conducted in small animals, such as mdx mice with Duchenne muscular dystrophy (DMD), which experience progressive muscle fiber degeneration due to continuous cycles of muscle myofiber degeneration and regeneration. The level of muscle regenerating myofibers and myofiber maturity in these animal models has been measured using dMHC [20,52,53]. Additionally, these studies showed that newly regenerating myofibers were marked by dMHC expression [52], whereas dMHC was practically undetectable in wild-type mice [53]. As the myofibers matured, dMHC was progressively replaced by adult myosin isoforms, resulting in a decrease in dMHC(+) myofibers [52,54]. The presence of regenerating myofibers, visible in the transverse muscle tissue sections within the injured area, is consistent with our sheep model. 

As previously reported [55], after a muscle injury, the cells involved in angiogenesis are chemo-attracted to the injury by these newly formed blood vessels, involving the recruitment and participation of blood vessel-derived cells in the repair process. These findings are related to the detection of α-SMA, a blood vessel marker, which was strongly increased in our sheep model, especially during the first 2 weeks. This important increase in the number of blood vessels invading the injured area, most likely coinciding with the muscle regeneration phase around 7–14 days after injury, would demonstrate an increase in blood and nutrient supply to the injured area during the muscle healing process. Increased vascular supply during muscle injury may also help to transport various cell types, such as inflammatory cells, to the muscle-injured area. Immune cells are involved in the modulation of skeletal muscle regeneration, and macrophages derived from blood monocytes are recruited to the injury site, where they secrete large amounts of inflammatory cytokines such as TNF-α, IL-6, and IL-1β [56]. In our sheep model, we detected the presence of CD68(+) macrophages throughout the muscle injury and healing process, especially during the first week after injury. Macrophages are involved in the whole process of muscle injury and repair. First, inflammatory macrophages (M1 subtype) participate in removing necrotic tissue; next, anti-inflammatory (M2 subtype) macrophages are associated with myogenic differentiation and tissue repair, and they are also present in the normal steady-state muscle, maintaining tissue homeostasis [57,58]. Previous studies in small animal models have shown that a prompt increase in the number of M1 macrophages after muscle injury promotes muscle healing, reduces the degree of fibrosis, and accelerates myofiber repair [59,60]. This suggests that macrophages play a pleiotropic role, participating not only in the inflammatory process, but also in angiogenesis, ECM remodeling, and in the muscle regeneration process [61]. 

Through MRI analysis, we could assess the injury at all time points in our sheep model and compare the results with those observed in human clinics. MRI and US have become highly valuable techniques and are widely used for assessing muscle injuries in athletes. In addition, there is good correlation between the two techniques [33,62]. MRI is considered the gold standard imaging method for evaluating muscle structure because of its capacity to provide high-quality contrast and resolution in visualizing soft tissues, making it particularly well-suited for use in cases where muscle injuries are clinically suspected [63]. In vivo MRI in the sheep model has mostly been used for the study of spinal muscles [64]; however, in this study we have used the newly developed sheep model to evaluate for the first time the longitudinal evolution of muscle injury in the gastrocnemius muscle by advanced 3 T MRI clinical imaging. We have observed partial rupture of the muscle fibers and presence of the classic feathery pattern, characteristic of this type of injury, which is produced by intramuscular edema. Intramuscular edema in the injured sheep muscle was detectable 3 days after injury and decreased over time, being only slightly detectable 4 weeks after injury. The healing process of these injuries described in human clinics takes approximately 12 weeks, with a decrease in edema during the first 2 weeks; however, the appearance of any interstitial edema on follow-up MRI should guide the recovery, training period, and return to play of the athlete [65]. In other animal studies performed in rodents, MRI analysis has been used to evaluate the extent of the muscle injury and has shown areas of edema that gradually decreased over time and with the muscle morphology substantially restored by the fifth day after the injury [25]. 

The results of our study could have broader implications for the field of skeletal muscle injury research by providing a more comprehensive understanding of muscle injury progression and prospective targets for intervention. In addition, the development of this sheep model opens up new avenues for translational research, with promising clinical applications. The model we developed has the capacity to advance therapeutic strategies and contribute to the development of targeted treatments for skeletal muscle injury, including the use of platelet-rich plasma injections [66], growth factor [67] or cell therapies [59]. Additionally, several pharmacotherapies to reduce fibrosis are currently being tested at both preclinical and clinical stages [68].

Our study has some limitations. Firstly, the sample size was limited, which may affect the generalizability of our findings and the robustness of the statistical analysis. However, this decision was based on the principles of the 3Rs (refinement, reduction, and replacement). Additionally, the study was limited to a one-month timeframe. Monitoring the muscle regeneration process in this animal model for a longer period could have been beneficial to provide more comprehensive data for comparison with human patients. However, it is critical to treat muscle injuries promptly, and the first month after injury represents the optimal therapeutic time for muscle injury management.

## 4. Materials and Methods

### 4.1. Animals

Twelve male sheep (Ripollese breed) aged 9–12 months were used as large animal models. Water and food were provided ad libitum throughout the experiments, except for a 16-h fasting period before the surgical intervention. All procedures were conducted under the supervision of specialized veterinarians, following Spanish (Real Decreto 53/2013) and European (2010/63/UE) regulations and were approved by the Departament d’Agricultura, Ramaderia, Pesca, Alimentació i Medi Natural of the Catalan Government, under procedure number 9939.

The animals were randomly divided into 4 groups (N = 3 for each time point) following the time points at which euthanasia was carried out (3, 7, 14, and 28 days post-injury). In all animals, the contralateral gastrocnemius muscle was left untreated and used as a healthy control.

### 4.2. Surgically Induced Skeletal Muscle Injury in a Sheep Model

A fentanyl transdermal patch (Durogesic^®^ Matrix, Janssen Pharmaceuticals, Titusville, NJ, USA) was applied to the forearm of each animal 24 h prior to the intervention at a dose of 2.5–5 µg/kg for analgesia. The animals were fasted from 5:00 p.m. the day before the intervention. On the day of the intervention, midazolam (Midazolam, B. Braun, Melsungen, Germany) was administered intramuscularly (IM) at a dose of 0.5 mg/kg for sedation. A catheter was inserted into the cephalic vein to administer 4 mg/kg of propofol (Propofol, Baxter International Inc., Deerfield, IL, USA) for anesthetic induction. During the intervention, the animal’s head was extended to prevent the accumulation of secretions, and 100% oxygen was administered through an anesthetic mask with 2–3% sevoflurane (Sevorane^®^, AbbVie, North Chicago, IL, USA). Additionally, fluid therapy was administered with Ringer lactate (Fresenius Kabi, Bad Homburg, Germany) at a rate of 2–3 mL/kg/h. Monitoring involved checking various parameters, including body temperature, heart rate, electrocardiogram, pulse oximetry, and invasive blood pressure.

To induce the traumatic skeletal muscle lesion in the sheep’s gastrocnemius muscle, the animals were positioned in the right lateral decubitus position. After cleaning and disinfecting the area, local anesthesia was administered using lidocaine (EMLA, AstraZeneca, Södertälje, Sweden). An ultrasound-guided transverse biopsy was used to perform the skeletal muscle injury at the myotendinous junction level of the medial gastrocnemius muscle in the right leg [30]. US procedures were performed using a Mindray M7 device and an L14-6Ns transducer (Mindray, Shenzhen, China), with a frequency range of 6 to 14 MHz. The muscle injury was generated using a 4 mm diameter 12 G biopsy needle (Bard^®^ Monopty^®^ Disposable Core Biopsy Instrument, Bard Biopsy Systems, Bard Peripheral Vascular Inc., Tempe, AZ, USA). For antibiotic prophylaxis, all animals were given cefazolin (Zepilen 1 g, Medochemie, Limassol, Cyprus) intravenously (IV) at a dose of 22 mg/kg during anesthetic induction. Postoperatively, amoxicillin (Amoxoil retard, Syva, Leon, Spain) was injected IM at a dose of 15 mg/kg and aluminum spray was applied to the injured area as an external antiseptic wound treatment. All animals returned to the housing area on their own feet and were allowed to move freely. Careful veterinary monitoring was implemented to ensure the welfare of the animals after the induction of the muscle injury, and regular examinations showed no signs of pain or morbidity.

### 4.3. Magnetic Resonance Imaging Analysis of the Longitudinal Evolution of Skeletal Muscle Injury

In vivo MRI studies (N = 3) were conducted 24 h after the surgically induced injury procedure, and at 3, 7, 14, and 28 days post-injury. The MRI scans were performed at the MRI facilities of the Comparative Medicine and Bioimage Center of Catalonia (CMCiB, Germans Trias i Pujol Research Institute (IGTP), Badalona, Spain), using a Vantage Galan MRI 3 T scanner (Canon Medical Systems, Otawara, Japan) for the image acquisitions. The MRI protocol involved acquiring T1-weighted images in the axial (AX), sagittal (SAG), and coronal (COR) planes (FOV: AX 100 × 180 mm, COR 120 × 100 mm, SAG 150 × 160 mm; matrix size: AX 192 × 320 mm; COR 208 × 192 mm; SAG 288 × 288 mm; TR: AX 1319 ms, COR 1309 ms, SAG 1282 ms; TE: 8.5 ms; slice thickness: 2 mm; gap: AX 0.5 mm, COR 0.6 mm, SAG 0.6 mm; in-plane resolution: 0.26 × 0.26 mm; nº of slices: AX 35, COR 45, SAG 34; acquisition time: AX 7:05 min, COR 2:51 min, SAG 6:12 min), a T2-weighted image in the same three planes (FOV: AX 100 × 150 mm, COR 120 × 100 mm, SAG 160 × 150 mm; matrix size: AX 320 × 320 mm; COR 240 × 192 mm; SAG 320 × 288 mm; TR: AX 5002 ms, COR 9151 ms, SAG 6853 ms; TE: 8.8 ms; slice thickness: 2 mm; gap: AX 0.5 mm, COR and SAG 0.6 mm; in-plane resolution: AX 0.24 × 0.24 mm, COR and SAG 0.25 × 0.25 mm; nº of slices: AX 35, COR 45, SAG 34; acquisition time: AX 7:05 min, COR 2:51 min, SAG 6:12 min), and a fat-suppressed PD-weighted image that was acquired in the AX, COR, SAG, and oblique (OB) planes (FOV: AX 100 × 180 mm, COR 120 × 90 mm, AX 150 × 160 mm, OB 150 × 150 mm; matrix size: AX 176 × 256 mm, COR 176 × 128 mm, SAG 240 × 224 mm, OB 272 × 256 mm; TR: AX 4714 ms, COR 4500 ms, SAG 4580 ms, OB 5000 ms; TE: 48 ms; slice thickness: 2 mm; gap: AX 0.5 mm, COR and SAG 0.6 mm, OB 0 mm; in-plane resolution: AX 0.28 × 0.28 mm, COR 0.34 × 0.34 mm, SAG 0.33 × 0.33 mm, OB 0.28 × 0.28 mm; nº of slices: AX 35, COR 45, SAG 34, OB 7; acquisition time: AX 5:49 min, COR 6:54 min, SAG 5:39 min, OB 3:05 min). Additionally, a DTI sequence was applied in the AX plane (30 direction; b = 800 s/mm^2^; FOV: 240 × 240 mm; matrix size: 128 × 128 mm; TR: 2750 ms; TE: 75 ms; slice thickness: 5 mm; gap: 1 mm; in-plane resolution: 0.94 × 0.94 mm; nº of slices: 26; acquisition time: 5:41 min).

On the days of the imaging tests, the animals were sedated with 0.5 mg/kg of midazolam and 4 mg/kg of ketamine (Ketolar 50 mg/mL, Parke-Davis S.L., Madrid, Spain) administered IM in the housing area. The cephalic vein was cannulated prior to their arrival at the clinical imaging area. Upon arrival, propofol (4 mg/kg) was administered for anesthetic induction before endotracheal intubation. Inhalation anesthesia with mechanical ventilation was initiated and maintained with inspired O_2_ fractions of 50% oxygen and capnography monitoring. To prevent ruminal tympany and minimize swelling caused by the accumulation of free gases in the rumen, an orogastric tube was inserted. Total intravenous anesthesia (TIVA) was administered continuously via an infusion pump at a dose of 11–14 mg/kg/h of propofol.

### 4.4. Histology and Immunofluorescence Analysis of Skeletal Muscle Injury

Animals (N = 3 for each time point) were euthanized after sedation with 0.5 mg/kg of midazolam and 4 mg/kg of propofol by an overdose of 200 mg/kg sodium thiopental (Tiobarbital 1 g, B. Braun, Melsungen, Germany) at 3, 7, 14, and 28 days after the injury. The gastrocnemius muscles were excised and immediately frozen in 2-methylbutane (Panreac Química S.L.U, Castellar del Vallès, Spain), which had been pre-cooled in liquid nitrogen, and stored at −80 °C until analysis. The frozen medial gastrocnemius muscles were transversely sectioned (10 µm thick) using a Leica CM3050 cryotome (Leica Microsystems, Wetzlar, Germany) at a temperature of below −20 °C and mounted on Polylisine^TM^ glass slides (VWR, Radnor, PA, USA). Consecutive frozen muscle sections were stored at −20 °C and used for subsequent histological and immunofluorescence analyses.

For the histological analysis, cryosectioned sheep skeletal muscle sections were stained with hematoxylin and eosin (1 min of hematoxylin and 15 s of eosin), washed in distilled water, dehydrated with graded ethanol solutions (ethanol 50%, ethanol 70% twice, ethanol 90%, and ethanol 100% twice, for 1 min each) and cleared in xylene (5 s). After air-drying for 5 min, the slides were mounted with DPX mounting medium (Sigma-Aldrich, St. Louis, MO, USA) and a coverslip (Menzel-Gläser^TM^, Thermo Fisher Scientific, Waltham, MA, USA). Harris’s hematoxylin solution was purchased from QCA (Química Clínica Aplicada S.A., Tarragona, Spain), and the eosin solution was prepared by dissolving 0.5 g of Eosin Yellowish (Panreac Química S.L.U, Castellar del Vallès, Spain) in 100 mL of distilled water with 200 μL of glacial acetic acid (VWR, Radnor, PA, USA). Ethanol absolute and xylene were obtained from Panreac (Panreac Química S.L.U, Castellar del Vallès, Spain). The histological samples were evaluated using a BX61 microscope (Olympus, Tokyo, Japan), a DP72 camera (Olympus, Tokyo, Japan), and CellSens^®^ Digital Imaging software (version 1.9, Olympus, Tokyo, Japan) for the visualization of muscle tissue samples to assess injury progression.

For the immunofluorescence analysis, cryosectioned sheep skeletal muscle sections were fixed in cold (−20 °C) acetone (Química Clínica Aplicada S.A., Tarragona, Spain) for 10 min, air-dried for 5 min, and blocked in phosphate-buffered saline (PBS; VWR, Radnor, PA, USA) 1x containing 3% bovine serum albumin (BSA; Biowest, Nuaillé, France) for 10 min at room temperature. Then, the muscle sections were incubated overnight with primary antibodies diluted 1:100 in PBS 1x + 3% BSA in a dark, humid chamber at 4 °C. The primary antibodies used were against rabbit anti-collagen I (ab34710, Abcam, Cambridge, UK), mouse anti-α-smooth muscle actin, α-SMA (A2547, Sigma-Aldrich, Merck Life Science S.L.U., Madrid, Spain), rabbit anti-CD68 (D4B9C) XP^®^ (#76437, Cell Signaling Technology, Inc., Danvers, MA, USA), and rabbit anti-Myosin heavy chain 3 (8087, ProSci Inc., Poway, CA, USA). The samples were washed 3 times each in PBS 1x and then incubated with the secondary antibodies Alexa Fluor^TM^ 568 donkey anti-mouse (A10037, Life Technologies, Carlsbad, CA, USA), Alexa Fluor^TM^ 568 donkey anti-rabbit (A10042, Life Technologies, Carlsbad, CA, USA), or Alexa Fluor^TM^ 488 donkey anti-rabbit (A21206, Life Technologies, Carlsbad, CA, USA), diluted 1:1000 in PBS 1x + 3% BSA in a dark, humid chamber for 1 h at room temperature. Finally, the samples were washed 3 times with PBS 1x and mounted using Fluoromount-G^TM^ with DAPI (Invitrogen, Waltham, MA, USA). Fluorescence was then evaluated using a BX61 microscope (Olympus, Tokyo, Japan) equipped with a DP72 camera (Olympus, Tokyo, Japan) and CellSens^®^ Digital Imaging software (version 1.9, Olympus, Tokyo, Japan). The images were measured using Fiji software (version 2.14.0, U. S. National Institutes of Health, Bethesda, MD, USA) to evaluate the density area of collagen I, dMHC, α-SMA, and CD68 in the muscle tissue samples. The values presented are an average of 6 to 8 images from each muscle sample within each study group, covering the entire injured area. Quantitative immunohistochemical analysis of the different biomarkers is presented as area density, which refers to the percentage of positive area for each marker relative to the total area of the image.

### 4.5. Statistical Analysis

Statistical analysis was performed with IBM SPSS Statistics version 26.0 (IBM, Armonk, NY, USA). Due to the small sample size, normality and homoscedasticity analyses of the data were not performed. As a result, nonparametric Mann–Whitney U tests were performed directly to assess statistical significance between injured and control samples for all variables. Differences were considered significant with a *p*-value less than 0.05. 

Additionally, nonparametric Kruskal–Wallis tests were performed directly to assess statistical significance for injured samples between the different time points (3, 7, 14, and 28 days post-injury) for all variables. The post hoc Dunn’s test was performed for multiple comparisons, and the Bonferroni correction was applied to control for type I errors. Differences were considered significant with an adjusted *p*-value less than 0.01.

The data are presented as mean ± SD for all the studied variables.

## 5. Conclusions

In summary, this study introduces a novel animal model for inducing skeletal muscle injury in sheep. The injury is generated through ultrasound-guided transverse biopsy at the myotendinous junction of the medial gastrocnemius muscle in a highly reproducible, fast, and simple manner. This sheep model allows for the evaluation of injury and regeneration processes in muscle and resembles several aspects of skeletal muscle injuries observed in human clinics. These features include the formation of fibrotic scar tissue, neoangiogenesis, inflammation, muscle regeneration, and the appearance of intramuscular edema in the injured area. In addition, the use of a minimally invasive and highly reproducible approach could contribute to the development of future models to replicate skeletal muscle injuries and explore novel targeted treatments. This new in vivo sheep model could represent a valuable tool for testing innovative therapeutic approaches to enhance muscle regeneration.

## Data Availability

Data are contained within the article or in the Supplementary Materials.

## Supplementary Materials

The following supporting information can be downloaded at https://www.mdpi.com/article/10.3390/ijms25115612/s1.
